# Lexical Processing in Deaf Readers: An fMRI Investigation of Reading Proficiency

**DOI:** 10.1371/journal.pone.0054696

**Published:** 2013-01-24

**Authors:** David P. Corina, Laurel A. Lawyer, Peter Hauser, Elizabeth Hirshorn

**Affiliations:** 1 Center for Mind and Brain, University of California Davis, Davis, California, United States of America; 2 Department of Research and Teacher Education, Rochester Institute of Technology, National Technical Institute for the Deaf, Rochester, New York, United States of America; 3 Department of Brain and Cognitive Sciences, University of Rochester, Rochester, New York, United States of America; University of Leuven, Belgium

## Abstract

Individuals with significant hearing loss often fail to attain competency in reading orthographic scripts which encode the sound properties of spoken language. Nevertheless, some profoundly deaf individuals do learn to read at age-appropriate levels. The question of what differentiates proficient deaf readers from less-proficient readers is poorly understood but topical, as efforts to develop appropriate and effective interventions are needed. This study uses functional magnetic resonance imaging (fMRI) to examine brain activation in deaf readers (N = 21), comparing proficient (N = 11) and less proficient (N = 10) readers’ performance in a widely used test of implicit reading. Proficient deaf readers activated left inferior frontal gyrus and left middle and superior temporal gyrus in a pattern that is consistent with regions reported in hearing readers. In contrast, the less-proficient readers exhibited a pattern of response characterized by inferior and middle frontal lobe activation (right>left) which bears some similarity to areas reported in studies of logographic reading, raising the possibility that these individuals are using a qualitatively different mode of orthographic processing than is traditionally observed in hearing individuals reading sound-based scripts. The evaluation of proficient and less-proficient readers points to different modes of processing printed English words. Importantly, these preliminary findings allow us to begin to establish the impact of linguistic and educational factors on the neural systems that underlie reading achievement in profoundly deaf individuals.

## Introduction

The orthographic representation of English encodes relationships between the sound-based properties of English words and conventionalized graphemic forms. As profoundly -deaf individuals often lack the ability to hear word forms of spoken English, a deaf learner’s ability to master the sound-form mappings is often hampered. Though this mapping in English is not fully transparent, decades of research with normally hearing children indicates that the appreciation of the relationship between visual symbols and the sounds these visual forms represent is often highly predictive of reading success [1]–[3]. However, while some profoundly deaf individuals do learn to read and process written English at levels comparable to their normally hearing peers, little is known about how these readers ultimately succeed in this task. In this study, we compare proficient deaf readers and less-proficient deaf readers in an attempt to characterize the patterns of brain activity that may differentiate these understudied groups.

Although opinions differ significantly [4], [5], two prevailing hypotheses about how deaf readers attain reading success can be identified. The most common assumption is that successful deaf readers are those who have obtained some degree of phonological awareness of English that is sufficient to provide a consistent mapping between visual letters and English words [6]. Support for this phonological hypothesis comes from the finding that, despite profound hearing loss, some deaf individuals obtain above-chance performance on English-based phonological tasks such as rhyming, even when orthographic similarity does not provide clues to sound-based similarity [7]–[13]. As might be expected, some of these studies have also reported a relationship between English phonological skills and reading [10], [13], [14], but see also [15]. It is often assumed in these cases that the successful deaf reader is in fact making use of auditory, articulatory, and perhaps visual properties of English phonology in forming relationships between print and meaning. Though not a necessary condition of the phonological hypothesis, it is reasonable to assume that these skilled deaf readers engage largely similar neural regions during reading as their hearing counterparts [16]. The assumption here is that while there may be subtle differences due to the distribution of effort required for relating visual symbols to lexical meaning, the fundamental cognitive processing is likely to be quite similar. Under this view, the successful deaf reader has simply been able to master the same mapping strategies utilized by hearing individuals, albeit at times with some compensatory strategies for inferring sound forms, such as a greater reliance on the articulatory and visual properties of oral English.

A second, though less well-developed, class of hypotheses posit that some successful deaf readers have mastered reading through a qualitatively different process than hearing individuals. Under one formulation, deaf individuals do not decompose English words into constituent sounds, but rather they are able to map directly between visual word forms and lexical-semantic representations [17], [18]. In some aspects, this hypothesis appears to be similar to the “whole-word” reading approach popularized by Goodman [19] and Smith [20]. However, proponents of this view suggest that the end result is a mapping not to English-based representations per se, but to semantic representations that underlie native-language abilities, which for many deaf individuals in the United States is American Sign Language [18], [21]. Proponents of this view suggest that deaf readers may resemble non-native (i.e. L2) speakers reading English, especially when early language preferences are based in signed languages. In support of this hypothesis, studies have shown that native (L1) competence in a sign language is a good predictor of success in English reading [22]–[25] and that there is evidence for activation of ASL forms during the processing of English word forms [26].

The idea that an alternative and non-phonologically based mapping between visual form and meaning exists is ensconced in the classic dual route models of reading, and in the more nuanced and complex implementations thereof [27]. The motivation for a non-phonological route for reading is based, in part, from consideration of the acquired dyslexias, specifically the syndrome known as deep dyslexia. Deep dyslexic readers are characterized by a constellation of reading errors which include impaired abilities to read non-words aloud, the presence of reading errors that are based in visual similarity (tribute → tribe; thing → thin), and additional unusual semantic errors (cart → horse; slope → snow). It is of further interest to note that deep dyslexics also make orthographic errors in spelling, which have been taken as evidence of lexical mediation across output domains (oral reading, writing, etc.) [28]. Collectively, these reading errors are thought to reflect the inability to make use of phoneme-to-grapheme routines and an over-reliance on the visual and semantic properties of print forms.

Studies of deaf readers’ spelling errors suggest that deaf subjects make more phonologically implausible errors than hearing counterparts (responsible → responbile; medicine → medince). While this may indicate a lack of appreciation of English phonotactics, they nevertheless tend to be orthographically legal. The sources of the observed spelling error patterns are difficult to categorize but likely reflect multiple constraints such as those on permissible syllable forms [29]. In their examination of deaf spelling errors, Olson and Caramazza [29] note that spelling patterns were not strongly predicted on the basis of purely visual-based frequency effects governing orthographic regularity as might be expected. For example, less common letter combinations are not replaced by more frequently occurring bigrams in these data. However, the visual-based frequency discounted by Olson and Caramazza is but one of many possible indications of an over-reliance on visual word form properties, and more work is needed to fully characterize the distribution and development of spelling errors in deaf subjects and how these patterns may reflect their processing of text.

Consideration of theories which suggest non-phonologically mediated routes of reading in deaf subjects have several implications for neural systems which may support successful reading in deaf signers. First, researchers have suggested that the non-phonological reading exhibited by deep dyslexics reflects a non-optimal right hemisphere visual language processing strategy [30], [31]. However, reading abilities observed in deaf readers have not implicated right hemisphere compensatory strategies. Second, theories which posit that deaf readers are making use of L1 ASL representations in their understanding of English print independently suggest differences in the neural representation of reading in deaf signers. For example, several studies have indicated that in hearing populations L2 reading engages the primary language areas associated with L1 written language processing, often to a greater extent [32], [33]. While research has shown that sign language processing calls upon left-hemisphere perisylvian regions in much the same way as spoken language processing [34], there remain subtle differences reported for ASL especially in the distribution of activations in posterior temporal-parietal regions. For example it has been shown that ASL processing in native signers recruits both the left and right posterior parietal regions [35]–[37], and that lesions to the right hemisphere can lead to impairments in the use of spatial-linguistic properties of ASL [34]. If skilled deaf readers are relying upon a mapping from English orthographic form to ASL semantic representations, it is reasonable to assume that this strategy would evoke activation in left hemisphere perisylvian language areas common to English and ASL, as well as regions believed to be unique to ASL processing, for example right hemisphere inferior parietal regions. Whether this same pattern of result would hold for less successful deaf readers is unclear.

Finally, whether successful deaf readers decompose English words into constituent sounds or process written English in a whole-word fashion, it is germane to consider the influence of the orthographic system itself. An emerging literature suggests that neural activation may be differentially distributed as a function of the form of script used [38], [39]. For example, research has shown that reading alphabetic scripts engenders more left-hemisphere fusiform processing whereas logographic writing systems like Chinese activate fusiform gyri bilaterally [40]. Moreover, studies have shown that the processing of logographic scripts evokes relatively greater activation in the left middle frontal gyrus (MidFG) while phonologically based scripts engender relatively greater activation in the left inferior frontal opercular region associated with phonological processing [41]. These script-based activation discrepancies are thought to reflect the differences in cognitive processing required for alphabetic words, which are predominantly read out by assembling fine-grained phonemic units (i.e., by assembled phonology) [42], [43], as opposed to the phonological codes resultant from logographic orthographies (i.e. addressed phonology) which are thought to arise after the visuo-orthographic information of the appropriate lexical candidate has been identified [41]. To the extent that orthographic forms for deaf readers are essentially non-canonically “assembled” they may show activation in neural regions which are more reflective of logographic scripts, which would give rise to differences in early temporal-ventral visual processing regions as well as differences in MidFG and opercular regions.

The consideration of experiential factors of deafness, coupled with theoretical models of reading in normal and disordered populations have led us to make several predictions regarding the neural regions which support the processing of orthographic word forms in English. To the extent that deaf readers are using some type of decomposition that entails aspects of phonological processing (whether auditory, visual or articulatory) we suggest these readers will show activations similar to that of hearing readers. Specifically we predict that we should find activation in left inferior frontal gyrus and left posterior-temporal regions. Conversely, under the premise that deaf readers are using qualitatively different means for understanding written words, we might expect neural activations that differ from hearing peers. These differences may manifest in several ways. First, to the extent that deaf readers mirror the strategies of acquired dyslexia (i.e. individuals who have lost the ability to use a phonologically mediated form for reading), we expect greater contributions of the right hemisphere. Second, if deaf readers are making reference to ASL representations during reading we expect English forms to activate L1 processing, which would include both left hemisphere perisylvian regions as well as language-specific right hemisphere activation, particularly in tempo-parietal regions. Finally, to the extent that deaf readers are using strategies akin to the reading of logographic scripts we expect to see activation in early temporal-ventral visual processing regions as well as differences between opercular and MidFG regions relative to what is traditionally reported for readers of alphabetic scripts. Finally, it is possible that a single modal pattern does not underlie both proficient and less-proficient deaf readers. Indeed relative degrees of proficiency may result in qualitatively different patterns between these subgroups of deaf readers.

In the following study, two groups of deaf readers were presented with English words and unrecognizable “false font” forms (see Methods for discussion and Supplementary materials for examples). In both cases, subjects were asked to analyze only visual characteristics of the stimuli by indicating whether each form had “tall” letters (those which ascended above the midline of the written forms). While both real English words and false fonts should recruit the same degree of low-level visual form processing, it is presumed that English words automatically and irrepressibly engage further lexical processing [44]. Characterizing the differences in processing between these two conditions allows for the identification of regions which contribute uniquely to reading above those responsible for decomposing complex orthographic stimuli. Furthermore, the patterns of activation between groups of proficient and less-proficient readers are compared, which sheds light on the processing strategies characteristic of these subgroups deaf readers.

## Results

The fMRI data reflect a summary statistics approach of random-effects models appropriate when inferences are to be applied to the entire population. An analysis of variance (ANOVA) was performed with Group (proficient vs. less-proficient readers) and Lexicality (words vs. false fonts) as factors. We examine significant main effects of lexicality reporting responses to words and false fonts. We then report a statistical group interaction that reveals neural regions that were more active in proficient readers than less-proficient readers during word reading compared to false fonts. A further investigation into the effects of reading proficiency is highlighted by consideration of a separate group analyses of proficient and less-proficient deaf readers (see Methods section for details). Please refer to Tables 1 & 2 for a list of activation foci and significance values for all comparisons.

**Table 1:** List of all activation foci in group analysis.

Group	Contrast	Cluster size	Coordinates	*t*	*z*	*p (uncorr.)*	Approximate locations
All subjects	Positive effect: Words	77	1, 10, 26	4.03	3.65	0.000	AC (BA 32)
		55	16, 39, 8	3.92	3.57	0.000	R MFG
		81	−2 −36 −43	3.92	3.57	0.000	L Cerebellum
		146	−53, 21, 26	3.65	3.35	0.000	L IFG (BA 45)
		103	−67, −36, 0	3.46	3.20	0.000	L MTG
		53	−13, 43, 4	3.37	3.13	0.001	L AC (BA 32)
	Positive effect: False fonts	45	34, −94, 8	3.33	3.10	0.001	R MOG (BA 18)
	Positive interaction: Proficient readers>Words>>Less-proficient readers>FFs	217	−10, 10, 44	3.77	3.45	0.000	L MFG
		208	−60, −15, 11	3.67	3.37	0.000	L STG (BA 22)
		56	−60, −40, 29	3.53	3.26	0.001	L SMG (BA40)
		67	62, −26, 26	3.19	2.98*	0.001	R SMG (BA40)
		76	−38, −54, −21	3.31	3.08	0.001	L Fusiform
		44	−49, −36, 0	3.21	3.00	0.001	L MTG (BA22)
		91	37, −44, −25	3.35	3.11	0.001	R Fusiform
		36	26, −69, 15	3.68	3.38	0.000	R Cuneus

AC = anterior cingulate; IFG = inferior frontal gyrus; MFG = medial frontal gyrus; MTG = middle temporal gyrus; MOG = middle occipital gyrus; STG = superior temporal gyrus; SMG = supramarginal gyru. Note “*” represents a Z value which falls below established criteria.

**Table 2:** List of all activation foci in the subgroup analyses.

Group	Contrast	Cluster size	Coordinates	*t*	*z*	*p (uncorr.)*	Approximate locations
Proficient readers	Words - false fonts	197	−20, 28, 40	6.03	3.83	0.000	L SFG (BA 8)
		19	16, 36, 51	5.55	3.67	0.000	R SFG (BA8)
		16	−38, −44, −21	5.76	3.74	0.000	L Fusiform
		16	62, −11, −7	5.35	3.59	0.000	R STG
		168	8, 10, 23	5.19	3.59	0.000	AC
		103	−67, −40, 0	4.33	3.18	0.001	L MTG
		28	66, −33, −3	4.67	3.33	0.000	R MTG
		73	8, −94, 15	5.12	3.51	0.000	R MOG/Cun (BA 18)
		26	−31, 36, −7	4.97	3.45	0.000	L IFG(BA 47)
		73	−53, 21, 15	4.97	3.45	0.000	L IFG (BA 44/45)
	False fonts - words	17	34, −94, 8	2.56	2.20*	0.014	R MOG (BA18)
Less-proficient readers	Words - false fonts	19	12, 39, 11	5.61	3.59	0.000	R Cingulate
		11	48, 21, 18	5.22	3.45	0.000	R IFG (BA 46/9)
		5	−53, 21, 26	4.11	3.01	0.001	L IFG (BA 45/9)
		5	12, −29, 15	5.08	3.40	0.000	Pulvinar
	False fonts - words	334	30, −33, −28	5.98	3.68	0.000	R Ant FusG
		170	−38, −90, −3	4.54	3.19	0.001	L Pos FusG

AC = anterior cingulate; FusG = fusiform gyrus; IFG = inferior frontal gyrus; MOG = middle occipital gyrus; MTG = middle temporal gyrus; SFG = superior frontal gyrus; STG = superior temporal gyrus.

### Group Results

Main effects for Group and Lexicality were significant (both F(1,32) = 9.09, p<0.005)). A Group×Lexicality interaction was also significant (F(1,38) = 7.35, p<0.01). Post hoc analyses were performed to analyze the contributions of individual groups and stimulus conditions. These results are presented below.

#### Positive effect: Words

An analysis of the positive effect of words (T(1,38) = 2.43, p<.01, k = 10) resulted in large regional activation in left inferior frontal gyrus (IFG), including the operculum (BA 45; peak at −53, 21, 26) with extension to the left middle frontal gyrus (BA8) as well as activation in the right medial frontal gyrus (BA10; peak 16, 39, 8). Prominent bilateral activation was also found in the anterior cingulate cortex (peaks at 1, 10, 26; −13, 43, 4),the left cerebellum, and left middle temporal gyrus (BA 22; peak −67, −36, 0).

The pattern of activity involving the left inferior frontal gyrus, left middle frontal gyrus, left middle temporal gyrus and the bilateral cingulate gyri is consistent with foci of reported in [44] which used the same task. However, in our data, inferior frontal gyrus activation was limited to the left hemisphere, while the activation reported in [44] is more ventral and bilateral.

The activation of the left frontal operculum has emerged as a consistent cluster in the meta-analysis of ortholinguistic activity reported in [45]. This region has been extensively described as being involved in aspects of semantic retrieval and selection processing [46]–[49]. In the context of the present experiment, this provides evidence that the implicit reading task is sufficient to engage aspects of semantic evaluation of word forms in deaf readers.

The activation of the anterior cingulate, a region commonly associated with error and conflict monitoring [50], [51], is greater in the context of monitoring for critical visual features in the presence of words rather than to meaningless false font word forms. In a fashion similar to the color Stroop task, automatic engagement of reading abilities in the task likely interferes with the attempt to make visual feature judgments. The suppression of the irrelevant dimension (in this case, reading) may result in greater anterior cingulate participation relative to the false font task, where no such implicit lexical activation is possible.

#### Positive effect: False fonts

The positive effect for false fonts (T(1,38) = 2.43, p<.01, k = 10) showed overall less activation, and revealed a pattern more consistent with the simple processing of visual stimuli. The comparison reveals right hemisphere activation in the inferior occipital extrastriate region (peak at 34, −94, 8) which has been associated with feature analysis, especially when contrasted with low-level baselines. This has been observed in a variety of domains, including object recognition [52], complex scene analysis [53], graphical form analysis [54], [55], and human action recognition [56].

#### Positive interaction: Group×Lexicality

In evaluating the interaction of Group by Lexicality (T(1,38) = 2.43, p<.01, k = 10), we find several regions that show significantly greater activation for the proficient deaf readers during word processing (relative to false fonts) compared to the less-proficient readers during word processing (relative to false fonts). Activations included left middle frontal gyrus (peak at −10, 10, 44), bilateral-fusiform gyrus (peaks at −38, −54, −21; 37, −44, −25), the left superior and middle temporal gyrus (peaks −60, −15, 11; −49, −36, 0), bilateral supramarginal gyrus (left>right; peaks at −60, −40, 29 and 62, −26, 26), left anterior cingulate, and the right cuneus. There were no regions that showed greater activation for the less-proficient readers compare to the proficient readers in evaluation of this interaction.

The fusiform activations found here are within the range of locations reported for the Visual Word Form Area (VWFA; mean −42, −57, −15) which Cohen et al. [57] situated at the ventral junction between the occipital and temporal lobes. Originally, the VWFA was characterized as a specifically left-hemisphere region responsible for prelexical processing specific to words or word-like stimuli. In its original description, the VWFA was considered a region with considerable plasticity, tuned to the orthographic regularities that constrain letter combinations during the acquisition of literacy [57], [58]. In subsequent work, the category-specificity of this region has been challenged [59], and research has further shown that a specialized visual analysis region may be seen in right hemisphere inferior temporal regions under some circumstances, as in the present case [60]–[62]. Current views suggest that one property of this region is in the participation of segmentation and classification of visually presented stimuli [45], an analysis which accords with our results.

It is important to note that this fusiform gyrus region showed greater activation in our proficient readers relative to the less-proficient readers. Several researchers have suggested that the responsivity of the VWFA is experience dependent [63]. Particularly relevant to our research is the observation that development of activation in VWFA is related to skill in reading, rather than maturation. Shaywitz and colleagues [64] reported that activation of the VWFA was positively correlated with standardized scores in grapheme–phoneme decoding ability. Such findings have been taken as evidence that successful mastery of grapheme–phoneme conversion (i.e. decoding) is a critical precursor to the development of the adult-like response properties of the VWFA [63].

A second region observed in this interaction was the left-hemisphere superior temporal gyrus (peak at −60, −15, 11). This region appears to lie proximal to temporal lobe region T1a (−56, −12, −3) reported in [45]. T1a is considered a “voice-specific area” [65], and is a common area of activation across phonological, semantic and syntactic judgments tasks suggesting this is a region for high-level linguistic integration. As discussed in [45], T1a is argued to have a dorsal component more associated with abstract phonological processing and a ventral part involved in the processing of intelligible words [45]. This characterization appears apt as our deaf subjects, who are unable to process auditory speech, may nevertheless be able to process abstract properties of phonological structure.

To further explore group differences at a finer level of detail, separate random-effects models were estimated using the existing contrasts for the proficient and less-proficient readers. The data from these models is reported below.

### Proficient Readers

#### Words – false fonts

**Figure 1:**
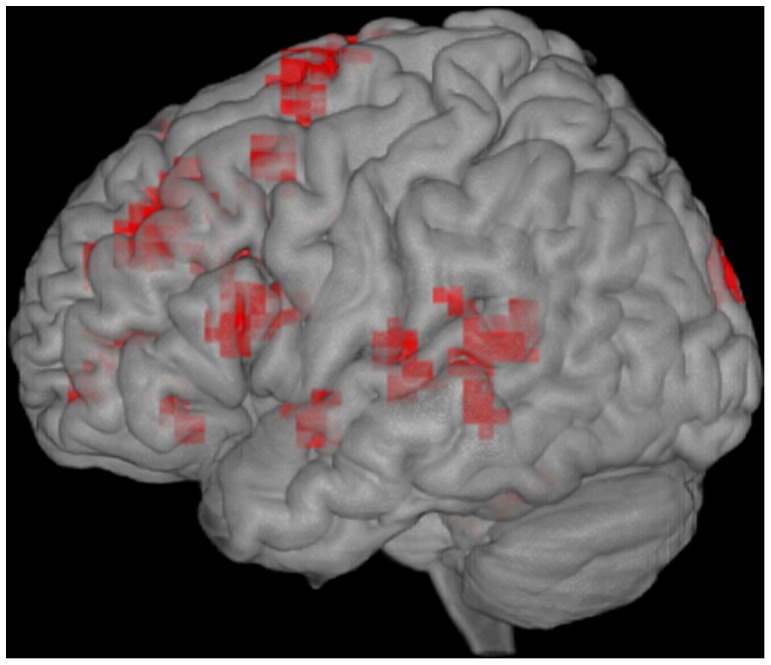
Activations in proficient deaf readers for words versus false fonts, p<.005.

For proficient readers, activation for word stimuli over false font stimuli (p<.005, k = 15) shows a stronger left-dominant pattern than in the previous full group results (see Figure 1). This includes left Broca’s area (peak −53, 21, 15) consistent with the activation found in the full group analysis as well as inferior opercular activation (BA 47, peak at −31, 36, −7). The opercular portion of Broca’s region has long been implicated in spoken language phonological tasks, including maintenance in phonological working memory, as well as retrieval, manipulation, and selection of phonological representations [46],[66]–[68]. Lexical access is known to rely in particular on Broca’s region in the left inferior frontal cortex, involving areas 44 and 45 [69]–[71]. Recent work utilizing cytoarchitectonic probability maps [72] suggests that area 45 supports lexical selection processes whereas area 44 is more involved in lexical access via the segmental route to reading. A number of studies of signed language processing have reliably found activation in the left IFG which further speaks to the modality independence of Broca’s region [36], [73], [74].

Proficient readers also exhibited bilateral activation of middle superior frontal gyrus (BA8, peaks −20, 28, 40; 16, 36, 51). A wide variety of functions have been assigned to this region, which has traditionally been associated with occulo-motor activity involving frontal eye fields, including activation of left BA 8, and secondary motor areas related to speech [75]. It is interesting to note that activation in BA 8 has been previously reported in studies of speech reading [76].

Another area of prominent activation was observed in bilateral middle temporal gyrus (MTG; peaks −67, −40, 0; 66, −33, −3) and adjacent right superior temporal sulcus (STS; peak 62, −11, −7). This posterior temporal region is commonly seen in tasks requiring word comprehension both in auditory and visual modalities [45]. As in the full group analysis, proficient readers showed robust activation in left posterior fusiform gyrus (−38, −44, −21) and anterior cingulate.

Finally, a small region in the right cuneus (peak 8, −95, 15) was observed during the reading of words relative to false fonts. Activation of the cuneus is associated with higher level visual processing including action recognition [77] and visual reading [55]. The involvement of this region may reflect activation of higher level properties of the visual word stimuli, and/or co-activation of action routines associated with sign language interpretation. Further work is needed to specify the role of this visual processing region in signing deaf readers.

Overall, the data from the proficient deaf readers suggests that these individuals are likely making use of neural regions for lexical recognition that are similar to those utilized by hearing individuals, in particular, involvement in the opercular region of the IFG which is suggestive of lexical selection, and the left-temporal lobe, often observed in studies of lexical semantics.

#### False fonts – words

The contrast between false fonts and words, produced no above threshold activation in our group of proficient readers. Activation at a reduced threshold was found only in the right middle occipital gyrus (peak 34, −94, 8), a region which has been reported in complex visual processing tasks, including reflecting on the physical appearances of famous persons [78] and visual memory for barcodes [79], suggesting a role of this region in evaluation of high level visual properties.

### Less-proficient Readers

#### Words – false fonts

**Figure 2:**
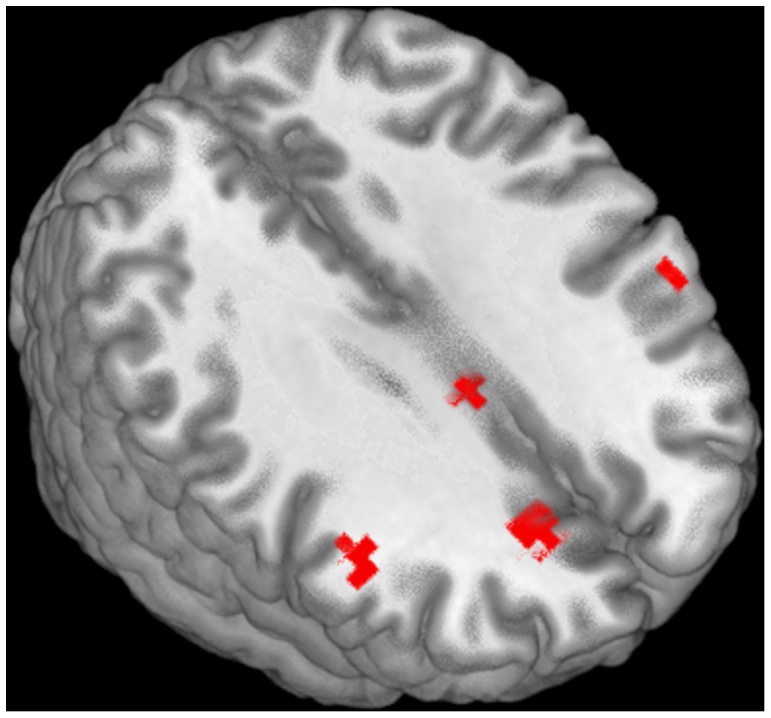
Activations in less-proficient deaf readers for words versus false fonts, p<.005.

Examination of the less-proficient deaf readers in the word versus false font contrasts (p<.005, k = 15) reveals a markedly different pattern of activation. Prominent activity was observed in the anterior cingulate (12, 39, 11) during this condition. Smaller clusters of activity (p<.005, k = 5) were located in and left and right middle and inferior frontal gyri BA46/9 (48, 21, 18), BA 44/9 (−53, 21, 26). This left inferior frontal gyrus activation lies dorsal to the IFG region observed in the proficient readers (see Figure 2). In this analysis, activation was also observed in the pulvinar (12, −29, 15).

The activation of the left middle frontal cortex in language processing has been repeatedly demonstrated in fMRI studies of logographic reading using Chinese. In particular, left middle frontal activation has been obtained in word generation [80], semantic judgment [81], homophone judgment (compared to fixation) [81], rhyme decision [81], and syllable decision tasks [82]. In a meta-review exploring activation during reading of alphabetic languages versus Chinese, Tan et al. [41] concluded that the left MFG is responsible for addressed phonology in Chinese reading. The involvement of the MFG in our less-proficient readers may indicate that for these deaf readers the implicit recognition of word forms engenders processing similar to that found in Chinese readers processing logographic scripts. Specifically, these deaf subjects could be processing English word forms as non-decomposable logographic-like forms analogous to Chinese.

There are, however, some differences between the current findings and those of Chinese logographic reading. First, the less-skilled deaf subjects’ activation was larger in the right hemisphere than left, whereas the results from the Chinese reading studies are clearly left hemisphere dominant. Second, we do not observe robust fusiform activation as might be expected (but see below).

While right hemisphere activation in the IFG and MFG has occasionally been reported in tasks of alphabetic reading, leading some to suggest different roles for phonology across the left and right hemispheres, there is little consensus and a general underreporting of right hemisphere effects in alphabetic reading. Pugh et al. [62] reported a correlation between right hemisphere IFG activation during phonological processing and regularity and word length effects. Regularity effects refer to the relatively slower ability to read words with irregular phoneme-to-grapheme correspondences (e.g. leaf versus deaf). In many models of reading, the reading of irregular words is suggested to place greater demands on lexical-semantic processing. Similarly, word length effects refer to the sensitivity of reading speed to the word length as measured by number of characters. One interpretation of these data would suggest that right hemisphere activation is evoked under conditions of effort, where traditional routes to successful reading are taxed. Thus, the presence of right hemisphere activation seen in the less-skilled deaf readers, encompassing both IFG (BA 46) and MFG (BA 46/9), may be a signature of an inefficient reading strategy. Additional data from reading skill matched hearing controls would be a useful means to further understand this unusual pattern of neural activation in less-proficient deaf readers.

#### False fonts – words

In the less-proficient readers, activation for false fonts over words (p<.005, k 15) is again dominated by visual processing. This contrast produced robust bilateral ventral fusiform (peak −38, −90, −3) and right fusiform activation (peak 30, −33, −28) The right fusiform region appears to be related to the left-hemisphere homologue of VWFA (−44, −51, −16), often reported in visual word processing tasks [57]. Here again it is interesting to note a hemispheric reversal of typically reported coordinates for the VWFA in the less-proficient readers.

The relatively large magnitude of visual fusiform activation during the false fonts over words comparison in the less proficient deaf readers compared to the proficient readers is noteworthy. This may be an indication that these less proficient readers are in fact making use of differential visual based analyses for these unusual orthographic false font forms to a greater extent than the more proficient readers. Note that a more expected pattern of left-hemisphere fusiform activation is present in the proficient deaf readers during the words over false font comparisons. Further work is needed to clarify these distributional differences in response to orthographic forms in these populations.

## Discussion

In our study, we speculated that neural imaging studies of deaf readers may result in a number of possible activation outcomes. Based both upon past research with deaf and hearing readers we outlined three possibilities. First, to the extent that deaf readers were using a phonologically based avenue for reading, we might expect neural activation to appear largely similar to hearing readers, specifically with activation in the left IFG, and the posterior and middle temporal regions. Second, we suggested that if deaf readers were making significant use of native language abilities, patterns of activation during English reading should map on to regions independently observed for ASL processing, notably left perisylvian regions and right temporal-parietal regions. Finally, to the extent that deaf readers were using a strategy that circumvented segmental analysis in favor of whole word form processing, we predicted involvement of the left hemisphere MidFG and activation in early temporal-ventral visual processing regions.

In the overall group analysis, we observed activation of neural regions that were largely similar to those reported previously using this implicit word recognition task. Prominent activation of left MidFG (BA 45) suggests that lexical-semantic processing is being engaged by deaf readers through implicit word recognition when contrasted with false fonts. The contrast of the false fonts relative to words resulted in activation of the right inferior occipital extrastriate regions consistent with a visual feature analysis of these novel and complex stimuli. These data are important as they extend previous findings in studies which have used this implicit reading task to investigate lexical processing in hearing adults, children, and dyslexic readers. Our data provide evidence that this implicit reading task engages neural regions associated with lexical-semantic and visual feature processing in deaf readers.

One concern raised in the present study is the relative lack of robust patterns of activation in the fMRI data. As seen in the whole group analysis, even with 21 subjects, significance cluster-level values for this reading measure do not often survive corrections for multiple comparisons. Careful inspection of our data suggests considerable variability in this population (resulting in less-robust significance values at the group level), and this heterogenity provides support for the notion that proficient and less-proficient deaf readers may be engaging in differing reading strategies which should be studied in their own right. As further knowledge regarding subgroups of deaf individuals who may exhibit differing reading processing strategies begins to accrue, one may expect more homogeneity to emerge. However, given the paucity of data in this research field, we are purposely taking a less conservative approach in the present paper. The reader should be aware of the limitations associated with this decision.

When deaf readers were divided in two groups on the basis of independently obtained reading levels, a particularly interesting finding emerged. In this analysis, we observed that proficient deaf readers activated the left IFG and the left STG in a pattern that is highly consistent with regions that have been reported in hearing readers. These data accord with the reports of Apracio et al. [16] and MacSweeney et al. [83] who also noted the prominent role of the left IFG in deaf readers during lexical decision and phonological judgment tasks. The MacSweeney et al. [83] report is of further interest due to the inclusion of a hearing dyslexic group in their study of pictorial rhyming. MacSweeny et al. [83] suggested that prominent left IFG activation is indicative of greater reliance on the articulatory component of speech during phonological processing when auditory processes are absent (deaf group) or impaired (dyslexic group). Thus, the brain appears to develop a similar solution to a processing problem that has different antecedents in these two populations. The differences in IFG foci between the deaf subjects (more ventral and superior) and the hearing dyslexics (more anterior and ventral) may reflect different degrees of reliance upon articulatory routines and lexical-semantic access in the service of these tasks.

Based upon the multiplicity of functions now attributed to Broca’s region (including its role in the mediation of sign language), one must exercise some caution in attributing left IFG activation to speech-articulatory processing. This point is underscored in the present study, where in contrast to the studies of Apracio et al. [16] and MacSweeney et al [83], which included a more heterogeneous mix of deaf subjects who used a variety of preferred communicative methods (i.e. oral speech, cued-speech and sign language (LSF & BSL)), had mixed sign-proficiency levels (only four of the seven participants in the MacSweeney et al [83] study had deaf parents and were assumed to be highly proficient signers and had attended oral-based educational school programs), the present study included only deaf subjects who were highly sign proficient (including 8–9 native signers in each group), reported ASL as their preferred mode of communication, and attended residential schools with sign-based instruction. Thus, based upon anatomy alone one cannot assume that the proficient deaf readers were using an oral approach to reading. Rather, given the reported role of BA 45 in aspects of language segmentation in the service of lexical-semantic processing, it seems plausible that these individuals were engaged in a more compositional approach of word recognition in this task relative to the less-skilled readers. Additional work is required to further tease apart how the pedagogical approaches to reading instruction and language competencies influence cognitive routines and the subsequent engagement of reading networks in skilled deaf readers.

The less-proficient readers exhibited a pattern of response characterized by bilateral middle frontal lobe activation (right>left) and a lack of temporal and/or parietal lobe activation in the word versus false font comparisons. This limited activation appears qualitatively different from that reported by Gizewskiet al. [84] who examined reading in German deaf signers and hearing non-signers. The adult deaf subjects had good to excellent knowledge of German Sign Language (DGS) but self-reported weak to moderate levels of reading ability. The deaf subjects exhibited a mix of etiologies, including prenatal and postnatal deafened individuals with delayed exposure to sign language that ranged from 0–6 years (mean 4.4 years). In this study, read narratives were compared to a baseline of meaningless character strings. The narrative paradigm produced widespread activation, including the left angular gyrus, bilateral occipitotemporal areas, and frontoparietal secondary motor areas in the deaf readers. In contrast, no activation of left temporal lobe (BA 21) was observed. Recall that in our study, the presence of left superior temporal lobe activation differentiated proficient from less-proficient deaf readers. For less-proficient deaf readers, we speculated that word recognition may reflect a less successful whole-word approach to word recognition, one which does not seem to fully engage regions that support a semantic analysis. Moreover, we suggested the middle frontal regions observed seemed similar to, though more medial than, the homologous left hemisphere regions characteristic of logographic reading in Chinese readers, further raising the possibility that these individual are using a qualitatively different mode of orthographic processing than is traditionally observed in hearing individuals reading alphabetic scripts. Finally the robust activation of bilateral temporal fusiform region in the less proficient group during the processing of false fonts relative to alphabetic strings suggest a different emphasis on visual form analysis of grapheme forms. Taken together, the comparisons of proficient and less proficient deaf readers have given an indication that qualitatively different neural processes may be engaged during single word reading.

To conclude, the implicit word reading task has proven useful in beginning to explicate the systems that deaf readers use during reading. Considerable heterogenity was found in the overall group results, supporting an evaluation of proficient and less-proficient readers which points to different modes of processing in deaf readers’ exposure to printed English words. Importantly, these preliminary findings allow us to begin to characterize the neural signatures related to linguistic and educational factors that underlie reading achievement in profoundly deaf individuals.

## Materials and Methods

### Participants

Twenty-one subjects participated in this study. Before beginning the experiment, written informed consent was acquired in accordance with the regulations of the Institutional Review Board of the University of California, Davis. Subjects completed background questionnaires which included items relevant to exposure to ASL. Each subject was further administered an assessment of their English reading comprehension (PIAT, Reading Comprehension Subtest) [85] after the fMRI session was complete. Median PIAT score was 73, and this served as the basis for placing the subjects into either above-median (proficient) or below-median (less-proficient) groups. Groups were designated for analysis only, and had no effect on the experiment in terms of task.

The proficient group had eleven subjects (8 female) with an average PIAT score of 84.55 (SD = 6.15) (mean grade equivalent: 8.7, mean age equivalent: 14). Age ranged from 19 to 46 (average 28.73, SD = 9.90). Ten subjects reported right-hand dominance; one subject was left handed. Nine subjects were native ASL users as indicated in the background questionnaire; the two remaining subjects reported first using ASL at age 13. The less-proficient group had ten subjects (7 female) with an average PIAT score of 60.80 (SD = 8.74) (mean grade equivalent: 4.0; mean age equivalent: 9.9). Age ranged from 19 to 45 (average 30.00, SD = 10.27) and nine subjects reported right-hand dominance; one subject was left-handed. Eight of the below-median subjects were native ASL users, with the remaining two subjects reporting using ASL from ages 8–9 and 13 respectively.

### Stimuli

**Figure 3:**
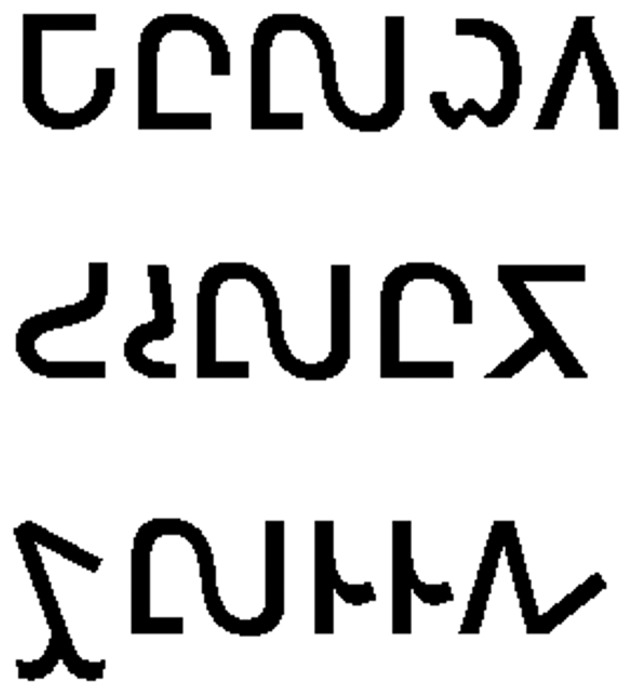
False fonts with no tall letters (corresponds to words “manor”, “ounce” and “groom”).

**Figure 4:**
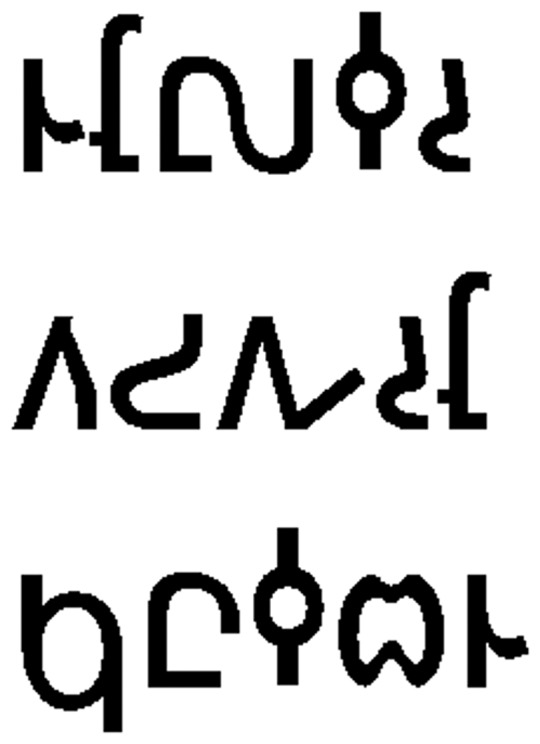
False fonts with tall letters (corresponds to words “stole”, “snort” and “pulse”).

Stimuli were composed of 40 English nouns and verbs (words) in standard lowercase orthography and 40 false font items (FF) both projected in black font on a white background. All word and FF items had five orthographic characters, and half of each set contained items with ‘tall’ letters (those which ascended above the midline of the word) as an experimental manipulation. FF items were created to match the original word stimuli in size, shape, distribution of ascending/descending letters, and overall orthographic frequency. These were created using items from previous implicit reading studies which utilize a font whose characters mimic English orthography in general composition, but are unrecognizable as known letters (see [44], [86] for discussion). See figures 3 and 4. A list of all word stimuli used in the experiment is provided in Supporting Information Stimulus Materials S1. A control condition which consisted of a black fixation cross on a white background was also included, and used as the baseline in analysis.

### Experimental Design and Image Acquisition

The experiment was comprised of alternating blocks of word and FF stimuli in two sets. Subjects were presented two alternating blocks of each stimulus type per set, which consisted of 10 unique stimulus items, randomly ordered. Each item was shown for 1 second, followed by 3 seconds of fixation. At the end of each block, the control condition was presented for 18 seconds. Completing the first set, subjects were allowed a break before beginning the second set. Both set order and block order within sets was counterbalanced across subjects.

In both word and FF conditions, subjects were asked to press a button to indicate whether the presented item contained a letter which ascended above the midline of the word (such as ‘t’, ‘h’, ‘l’, or ‘d’). All items required a yes/no button press response. Subjects were shown examples of word and FF items similar to those used in the task prior to scanning to ensure they understood the directions. None of the items used in training were included in the experiment. Each subject completed both sets, resulting in the presentation of 40 Word and FF items each. Each set took approximately 5 minutes to complete. Subjects also participated in a second study on ASL and gesture perception, discussed in a separate paper.

Imaging data was acquired on two Siemens Trio Tim 3T scanners located at the University of California, Davis Imaging Research Center in Sacramento, California and at the Rochester Center for Brain Imaging in Rochester, New York. A standard Siemens 8-channel head coil was employed in both locations, with added foam padding to minimize subject head movement during scanning. Four functional runs (two word/FF and two sign/gesture) and one structural image were acquired from each subject. Functional runs consisted of 89 volumes and were collected using a gradient echo EPI sequence (46 slices, thickness = 3.6 mm, TR = 3000 ms, TE = 30 ms, flip angle = 90-, FOV = 230 mm×230 mm, voxel size = 3.6 mm^3^). Functional volumes were aligned parallel to the anterior-posterior commissure (AC-PC line) and provided full brain coverage. The initial 8 fixation volumes acquired at the beginning of each run were discarded from analysis.

During scanning, stimuli were presented via a Digital Projection Mercury 5000HD projector. The experiment was back-projected onto a screen placed at the foot of the scanner bed. A mirror mounted to the head coil and angled at approximately 45 degrees allowed subjects to comfortably view stimuli from inside the scanner bore. Each subject verified their ability to see the stimuli and adjustments were made to mitigate eye strain. Following the acquisition of functional images, a high-resolution structural image covering the entire brain was acquired using an MPRAGE sequence (208 slices, thickness = 1 mm, TR = 1900 ms, TE = 3.06 ms, flip angle = 7-, FOV = 256 mm×256 mm, matrix = 256×256, voxel size = 1 mm^3^).

### Data Analysis

Data from all subjects was preprocessed before being submitted to statistical analysis using SPM8 (Welcome Department of Imaging Neuroscience). All volumes with large movement artifacts were removed from analysis. Remaining images were slice time corrected and realigned to each subject’s mean image. Both structural and functional images were coregistered to the mean image, and normalized to the MNI template to enable group comparisons. Functional images were smoothed with an 8 mm^3^ FWHM Gaussian kernel.

A random-effects statistical model was used to quantify BOLD effects. First-level condition-related changes in regional brain activity were first estimated for each participant according to the general linear model fitted with the parameters for each condition (words, false fonts, fixation) and each subject’s 6 realignment parameters included as regressors. Significant cerebral activations for the critical contrasts (Words-Fixation, False Font-Fixation) of interest were then examined at the second-level in SPM using a 2×2 analyses of variance (ANOVA) with factors of Group (Proficient vs. Less-proficient) and Lexically (Words vs. False Fonts). Positive interactions for each group were tested using post-hoc T-tests with significance level of p<.01 uncorrected, and a 10 voxel cluster. In addition, separate random-effects models were estimated in SPM for the contrasts (Words-False Fonts) and (False Fonts-Words) for the proficient and less-proficient subjects respectively. Given the smaller sample size, unless otherwise noted, these individual contrasts were evaluated at p<.005 uncorrected, 15 voxel clusters. Values reported in the tables 1 & 2 reflect activations which exceed Z≥3, p = .0013.

## Supporting Information

Stimulus Materials S1 (DOCX)
