# Supplementary material for: Lexical Processing in Deaf Readers: An fMRI Investigation of Reading Proficiency
Source: PLoS One. 2013 Jan 24;8(1):e54696. doi: 10.1371/journal.pone.0054696 (PMC3554651; doi:10.1371/journal.pone.0054696)
Supplement: Stimulus Materials S1 — (DOCX) [file pone.0054696.s001.docx]

Stimulus Materials S1

Stimulus List: Words, Set 1

Block 1 Block 2

gourd pearl

carob woven

sewer repay

heave chess

solve snort

ounce scout

vowel gypsy

cease manor

caste coble

sauce super

Stimulus List: Words, Set 2

Block 1 Block 2

sprue cozen

gamma stoop

dross grasp

cream verve

array pulse

squat fetch

quote groom

beast arose

stole alarm

parry marry
